# A bimodal image dataset for seed classification from the visible and near-infrared spectrum

**DOI:** 10.1038/s41597-025-05979-6

**Published:** 2025-10-08

**Authors:** Maksim Kukushkin, Martin Bogdan, Simon Goertz, Jan-Ole Callsen, Eric Oldenburg, Matthias Enders, Thomas Schmid

**Affiliations:** 1 Martin Luther University Halle-Wittenberg, Halle(Saale), 06108 Germany; 2 Leipzig University, Leipzig, 04109 Germany; 3 NPZ Innovation GmbH, Holtsee, 24363 Germany; 4 Norddeutsche Pflanzenzucht Hans-Georg Lembke KG, Hohenlieth-Hof 1, 24363 Holtsee, Germany; 5 Lancaster University in Leipzig, Leipzig, 04109 Germany

**Keywords:** Agriculture, Research data

## Abstract

The success of deep learning in image classification has been largely underpinned by large-scale datasets, such as ImageNet, which have significantly advanced multi-class classification for RGB and grayscale images. However, datasets that capture spectral information beyond the visible spectrum remain scarce, despite their high potential, especially in agriculture, medicine and remote sensing. To address this gap in the agricultural domain, we present a thoroughly curated bimodal seed image dataset comprising paired RGB and hyperspectral images for 10 plant species, making it one of the largest bimodal seed datasets available. We describe the methodology for data collection and preprocessing and benchmark several deep learning models on the dataset to evaluate their multi-class classification performance. By contributing a high-quality dataset, our manuscript offers a valuable resource for studying spectral, spatial and morphological properties of seeds, thereby opening new avenues for research and applications.

## Background & Summary

The significant advancements in state-of-the-art architectures over the past decade would not have been possible without the availability of large-scale datasets, which enabled the training of Convolutional Neural Networks (ConvNets) and Vision Transformers (ViTs). Some of the most impactful datasets include ImageNet^1^ for image classification, SA-1B^2^ for image segmentation, YouTube-8M^3^ for video classification and Kinetics-400M^4^ for action recognition. The large-scale datasets have established benchmarks crucial to advancing existing deep learning architectures and developing new ones.

In the context of seed classification, the diversity and quantity of available data was often insufficient. Previous datasets have frequently included a limited number of samples per class or low number of seed species in the dataset. For instance, Granitto *et al*. (2002)^5^ introduced the first seed dataset which consisted of 3,163 colour images of 53 seed species. In following years, Granitto *et al*. (2005)^6^ expanded the dataset to include images of 236 species which brought the total number of RGB seed images to 10,310. In their work, the authors trained a Multilayer Perceptron and a Naïve Bayes classifier. More recent study^7^ presented a dataset of 140 species with 47,696 RGB images. The authors evaluated the dataset’s performance using six different ConvNets. The latest work^8^ demonstrated an RGB seed image dataset consisting of 4,496 images which consisted of 88 species with an average of 50 samples per species, where each image was captured with a smartphone camera. A common limitation across these datasets is the relatively low number of images per species, which raises concerns about their suitability for training large deep learning models, such as ConvNets or ViTs and typically require vast amounts of data^1,9^. Refs. ^10,11^ provide more comprehensive overviews on published seed classification papers and the seed species datasets.

Recently, multimodal datasets have started to play a crucial role in advancing computer vision by enabling the fusion of imaging modalities from different sensors, which led to a more robust and thorough analysis of imaging data. Unlike unimodal datasets, which capture only a single aspect of imaging information, multimodal datasets integrate additional data sources such as RGB, motion, depth, hyperspectral, or LiDAR images. Recent studies reported increased accuracy for multiple scenarios by using modality fusion in diverse applications, including medical imaging, remote sensing and autonomous systems. For instance, in medical diagnostics, combining Magnetic Resonance Imaging (MRI), Computed Tomography (CT) and Positron Emission Tomography (PET) scans improves disease detection and localisation by leveraging the strengths of each modality^12–14^. In autonomous driving, the fusion of RGB, LiDAR and radar data provides more reliable object detection and depth estimation^15–18^. Similarly, hyperspectral and multispectral image fusion^19^ has proven effective in remote sensing applications^20^, enhancing vegetation^21^, mineral detection^22^ and environmental monitoring^23^. The ability to jointly learn representations from multiple modalities not only improves performance but also increases model robustness and generalisation to real-world scenarios. As sensor technologies continue to evolve, multimodal datasets will remain fundamental in pushing the boundaries of computer vision applications.

Despite the clear advantages of multimodal datasets, their main limitation is their availability. Incorporating additional modalities increases costs and requires significant time for data collection, calibration and annotation. Our bimodal seed image dataset (BiSID-5k)^24^ addresses the challenge by providing a high-quality dataset for seed classification. Unlike prior studies that relied on datasets with a limited number of samples, BiSID-5k^24^ encompasses 10 distinct seed species and extends beyond traditional RGB images by incorporating fine-grained hyperspectral imaging data. With 500 samples per species, BiSID-5k^24^ is, to the best of our knowledge, one of the largest datasets for seed classification. Moreover, it is the largest bimodal seed dataset containing both RGB and hyperspectral data. Furthermore, we experimentally demonstrate the effectiveness of hyperspectral modality for seed classification, showcasing its potential for feature extraction in this context. Ultimately, our study provides a comprehensive evaluation of hyperspectral feature extraction methods, contributing to the broader adoption of multimodal datasets in seed classification.

## Methods

### Challenge

In agricultural seed production, maintaining seed quality is a major challenge. This is driven not only by the need to meet customer expectations but also by mandatory international and national regulations. For instance, the International Seed Testing Association (ISTA) provides guidelines that many countries adopt to ensure conformity in seed testing. It is important to note that while ISTA provides international guidelines, individual countries may have own specific regulations^25–27^ that align with or differ from these standards. For example, the European Union (EU) enforces strict quality control standards, including the certification of seed lots before they can be sold^28^. Similar rules are valid in other countries e.g., Argentina^6^, Sweden^29^ or United States (US)^30^. Thus, seed producers are required to analyse and categorise harvested seeds to ensure compliance, a task that typically relies on trained human analysts. Due to the large number of weed species, which can potentially occur in a seed production, as well as the high variability of the seeds itself as a biological object a valid classification is quite challenging and need well trained human experts. This challenges of a high class number as well as high intra-class variability is further aggravated by partial low inter-class distinctness. This later challenge arise, as some weeds exhibit very similar seed properties like shape, size or colours of the seeds due to the close relationship of some species or a phenomenon known as *vavilivian mimicry*^31^. As commercial seed productions can comprise tons of seeds, a statistically sufficient and representative sample has to be analysed by human experts to comply with the legal frameworks of most countries. Therefore, a huge number of samples, containing a large number of single seeds must be screened by experts in the short timeframe of the harvesting season before sowing. For instance, in the context of oilseed rape in German seed certification, a sample of 100 g of seeds (20,000 seeds, assuming a thousand seed weight of 5 g) must be analysed and screened for seeds of other species and technical impurities^32^.

### Overall concept

**Fig. 1:**
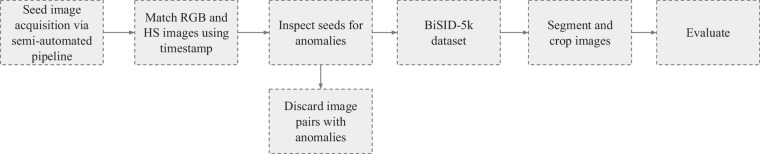
Overview of the pipeline for BiSID-5k dataset^24^ acquisition and preparation.

The overall concept of the dataset acquisition (see Fig. 1) was to represent seeds of European weed species which are frequently found in commercial oilseed rape (*Brassica napus* L.) seed productions. Ten of those species have been selected and single seed images has been acquired using a hyperspectral- as well as a RGB camera. This results in image pairs depicting the very same seed. In contrast to other studies^33,34^, which employed YOLO^35^-based approaches designed for detecting and classifying multiple seeds within a single image (e.g., entire seed lots), we chose a simpler approach—similar to that of^7,36^—in which each image contains exactly one seed. Finally, to account for the potential demand of neuronal networks for large and balanced training datasets, 500 single seeds per species where represented in the dataset. This results in a perfectly balanced labelled multimodal image dataset of 5,000 seeds covering 10 species and containing 10,000 images: 5,000 hyperspectral and 5,000 RGB images respectively.

#### Data acquisition

##### Custom seed tray

To obtain image data, seeds where individually placed on a single 30 mm  × 30 mm plate. Plates were produced using additive manufacturing, using Extrudr-Green-TEC Pro Filament - 1.75 mm, blue and an Raise3D Pro2 Plus 3D printer. Each plate has an 18 mm  × 18 mm square in the centre with raised and white marked walls. The size of the inner square is sufficient to accommodate all seed species under consideration, despite their significant variations in shape and size. Furthermore, an oval recess is placed inside the inner square, which optimises the central positioning of the seeds. In particular, this is intended to prevent seeds from touching the edge of the inner square. An Computer-Aided-Design (CAD) model of the plates can be obtained from the authors on request for scientific purposes.

##### Camera and lightning equipment

Two camera setups placed in an array were employed to acquire image data.

1. RGB images were acquired using a Raspberry Pi High Quality Camera (Sensor: Sony IMX477R, 12.3 MP) in combination with a CS/C mount lens (0.12-1.8x) distributed by Pimoroni Ltd. (Sheffield, United Kingdom). The distance between seeds and the lower end of the lens was 16 cm. Lighting was ensured using a white LED SMD ring light (70 mm diameter by HexaCube). The camera was controlled by the picamera2 package (https://github.com/raspberrypi/picamera2) using an exposure time of 5 ms and no auto white balance mode (AwbMode = False).
2. Hyperspectral image data captured using the Resonon (USA) Pika L 100121-220 model, covering wavelengths ranging from 380.96 nm to 1017.9 nm in the visible and near-infrared (VNIR) region of the electromagnetic spectrum, with a spectral resolution of 5 nm. This camera was used in combination with a Tele-Xenar 2.2/70MM Compact lens by Schneider-Kreuznach (Germany) and 4x halogen light bulbs (Osram Decostar 51 Pro, 35 Watts, 3000 Kelvin light colour, 36^∘^ opening angle) for lighting. The camera was calibrated using teflon target. Any other natural or artificial lighting, except the ones mentioned before were excluded. Distance between seeds and lower end of the lens was 14.5 cm. We employed the Resonon SDK (https://docs.resonon.com/API/html/index.html, Version 3.4.11) using 36 Hz framerate, 1 ms integration time and 0 Db gain for hyperspectral image acquisition.

##### Image acquisition process

**Fig. 2:**
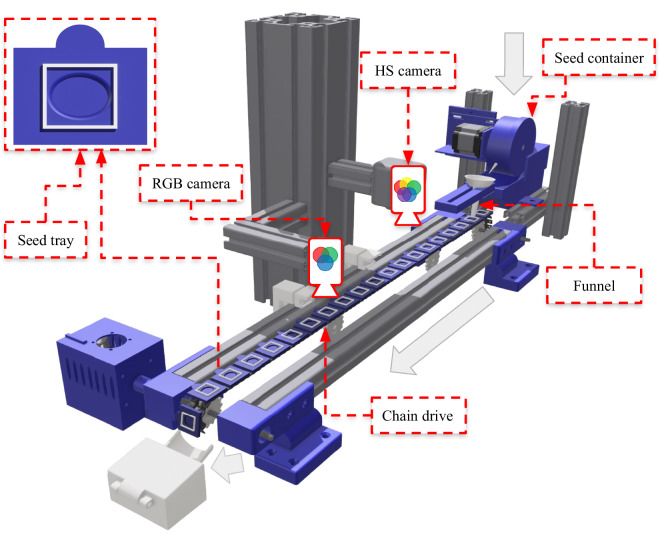
Semi-automatic data acquisition pipeline. The pipeline consists of a constant-speed chain drive, where each seed is placed in an individual seed tray. As the seed tray reaches the centre of the pipeline, hyperspectral and RGB cameras are triggered and capture a bimodal pair of hyperspectral and RGB images.

The single seeds, placed on the custom seed tray, are transported through the camera array using a constant-speed chain drive. Light barrier switches were used to separately trigger the RGB and hyperspectral cameras. Due to the fixed and known time-offset of both cameras, individual RGB- and hyperspectral image-pairs, which depict the same seed, are paired using the image capture timestamp of the respective files. The complete setup is shown in Fig. 2.

#### Data curation & collection

**Fig. 3:**
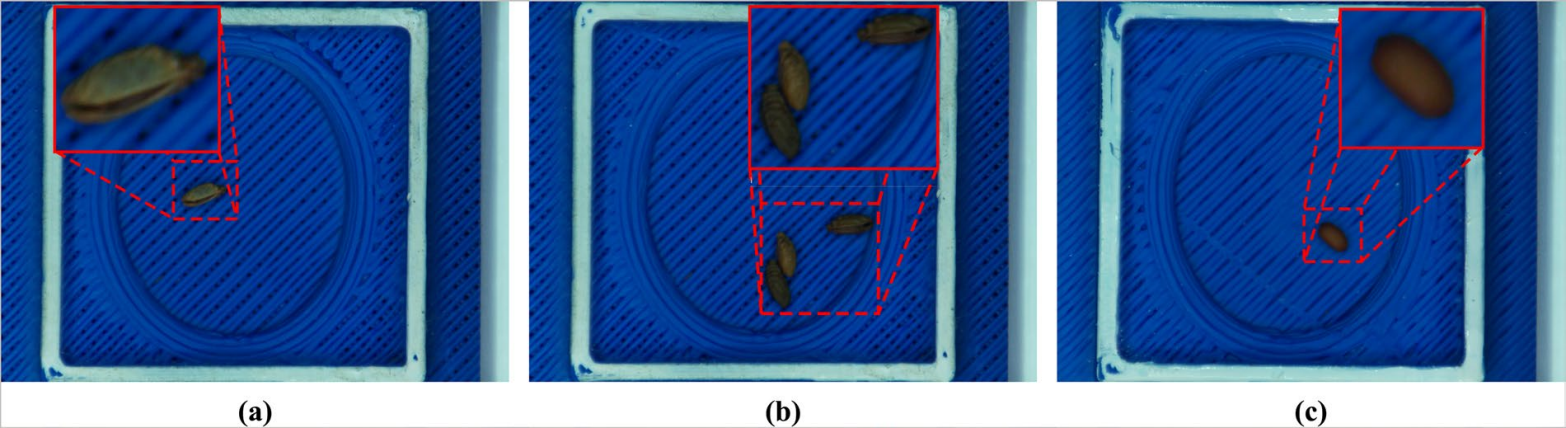
Examples of seed variation for the species *Geranium robertianum* L. (**a**–**c**): (**a**) seed image without any physical anomaly, (**b**) multiple seeds in a single image, (**c**) seed without a coat.

Harvesting, packaging and transporting of seeds can introduce physical anomalies, such as damage to the seeds or the absence of seed coats. These factors can complicate data analysis, as the lack of a seed coat, for instance, may alter the spectral properties of the seeds. Additionally, clumped seeds pose a challenge, as they may remain undetected during the semi-automatic image acquisition process and only be identified later. These issues can reduce the accuracy of data collection and subsequent analysis. Some of the aforementioned issues are depicted in Fig. 3.

To ensure a high quality data source for deep learning approaches, the dataset presented in this article was manually and visually inspected and subsequently filtered. Images which exhibit not exactly one seed of the desired species were excluded by a qualified seed analyst. Furthermore, very few seeds which differs significantly from the species seed phenotype were excluded too. This later case covers the aforementioned situations of damaged or dehulled seeds, as well as foreign seeds of plant species, which are represented in the seed stock in rare cases.

#### Preprocessing

**Fig. 4:**
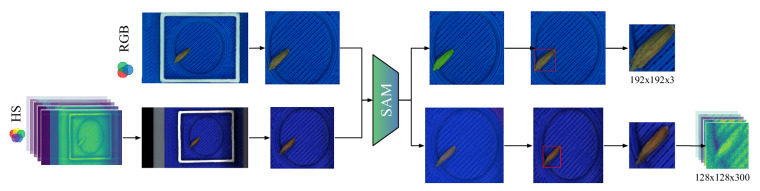
Example of image processing pipeline for BiSID-5k dataset^24^. The pipeline involves segmentation of both RGB and hyperspectral (HS) modalities. Initially, a white square box is detected in both modalities. The region containing the seed inside the white box is cropped. Subsequently, the Segment Anything Model (SAM)^2^ with a ViT-B backbone is applied to segment the image, isolating the object likely near the centre. Finally, a bounding box is drawn around the identified object and subsequently cropped. Notably, unlike the RGB modality, segmentation for the HS modality is performed on synthetic RGB images derived from the hyperspectral cubes. The segmentation results are extrapolated to the original hyperspectral image.

**Fig. 5:**
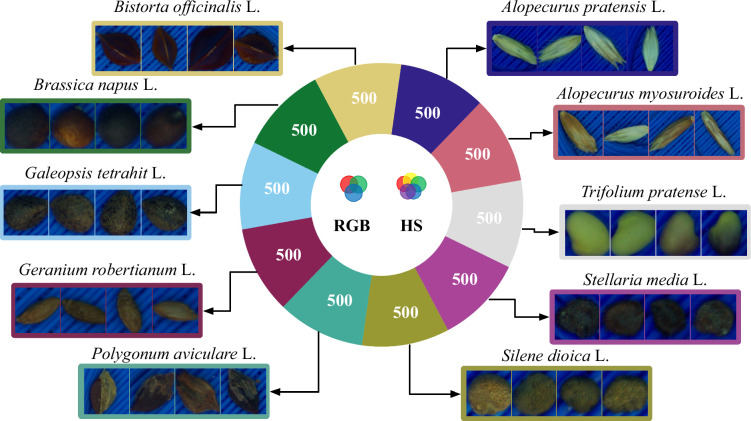
Overview of the BiSID-5k dataset. The BiSID-5k dataset^24^ consists of 10 plant seed species with 500 bimodal pairs per class. Each sample contains a pair of one hyperspectral and one RGB image, respectively.

**Fig. 6:**
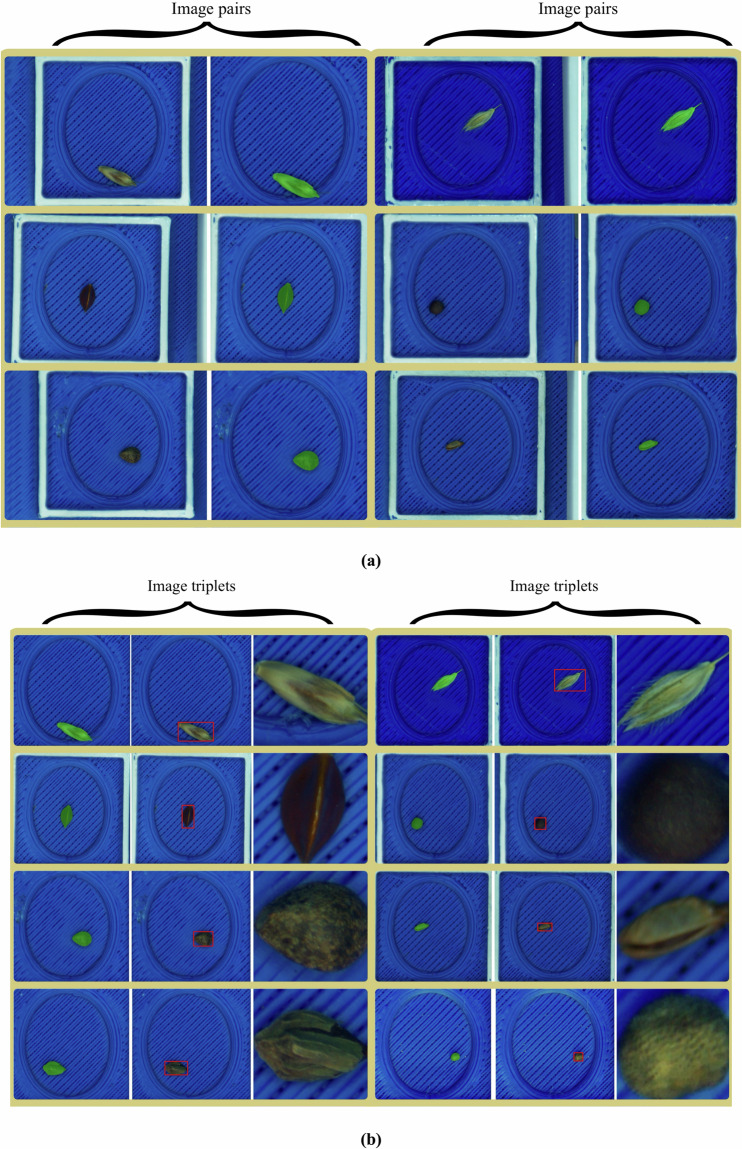
Seed segmentation results. Displayed are (**a**) 6 pairs of original seed images and their corresponding segmentation masks extracted using SAM^2^, (**b**) 8 triplets consisting of seed segmentation masks, seed bounding boxes and the corresponding cropped images.

The preprocessing of dataset images is straightforward and uniform across modalities. For both RGB and hyperspectral images, we employed the pretrained Segment Anything Model (SAM)^2^ with a ViT-B backbone, to segment and isolate the seeds from the white boxes of the seed tray. As SAM allows to use RGB images only (and not hyperspectral images), we generated synthetic RGB images from hyperspectral cubes to ensure compatibility. Specifically, we utilise the 640 nm waveband for the red, 550 nm for green and 459 nm for blue channels. After segmentation step, bounding boxes enclosing each seed were extracted for both modalities. Then, the images were resized to enable model training: RGB images were resized to dimensions of 192 × 192 × 3, while the hyperspectral cubes were resized to dimensions of 128 × 128 × 300. In these notation, the first two numbers represent spatial dimensions (height and width), while the last number indicates the number of channels (3 for RGB) or spectral bands (300 for hyperspectral). Finally, the entire process and the resulting images are presented in Figs. 4 and 5, respectively. Additional examples illustrating the results of each preprocessing step are shown in Fig. 6.

#### Dataset limitations

Collecting a representative dataset for each seed species is a very complex task, as seeds exhibit high intra-class variability due to many factors^11^. Below, we describe the most common ones.

##### Geographical and temporal variability

Seeds of the same species can exhibit significant variability depending on their geographical origin. For example, seeds harvested in southern France may display different characteristics compared to those collected in northern Germany. Moreover, this variability is not limited to geography; seeds collected from the same location may differ across years. These differences are primarily driven by regional climate conditions, such as the number of sunny days, droughts, or floods in specific years. In this study, the dataset does not account for geographical or temporal variability, as all seed samples for each species were sourced from a single region within a particular year.

In addition, the occurrence and distribution of foreign seed classes vary from seed lot to seed lot and depend on geographical location and harvest year. For instance, in a given seed lot, some foreign seed species may be completely absent, while others may be overrepresented. The balanced, equally distributed dataset presented in this paper cannot reflect these real-world seed-to-seed lot differences in foreign seed distributions. Depending on the application, potential users of the dataset can apply oversampling^37^ and dataset augmentation techniques (e.g., MixUp variants^38–40^) to better mimic real-world distributions.

##### Growth stage variability

Seeds from the same species can exhibit subtle variations in characteristics such as colour and texture, depending on the plant’s growth stage at the time of harvest. This study does not include metadata specifying the growth stage at which the seeds were collected, which could potentially limit the dataset’s representativeness.

##### Hyperspectral image data

The range of light (380.96 nm to 1017.9 nm) captured and represented in our dataset is limited, as hyperspectral cameras are commercially available which captures spectral image data up to 2.500 nm and beyond. Nevertheless, those systems are financially demanding and most often do not achieve a high spatial resolution.

##### RGB image data

As reported in the literature^41^, microscopic seed coat structures can be utilised to identify the respective plant species. These patterns can be recorded using, for instance, electron microscopy. Our RGB image data is limited in terms of spatial resolution as well as depth of field. Furthermore, only one side of each seed is imaged. Thus, it is evident that seeds exhibit discriminative features that are not fully accessible in our dataset due to limitations in the image recording techniques.

##### Sharp seed colour variability and preprocessing challenges

The high variability in seed appearance presents significant challenges for automated preprocessing, particularly during segmentation. Sharp discontinuities in colour and texture—arising from physical factors such as seed breakage, incomplete hulling, or surface damage—can cause segmentation algorithms to fail.

To overcome the limited representativity of seed datasets, future datasets should include a sufficient number of additional seed samples that address the mentioned factors affecting seed traits, e.g., shape, color, and spectral signature. Each sample should be accompanied by detailed metadata documenting the presence of these factors. Only in this way can we more comprehensively capture both inter-class and intra-class variability and ensure high representativity in future seed datasets.

## Data Records

The dataset is publicly available at 10.25532/OPARA-810 under the Creative Commons Attribution (CC BY) license. The BiSID-5k^24^ consists of 500 paired samples for each of the 10 species (see Table 1), organised in directories named after their respective species. Each sample is stored in a dedicated sub-directory with a unique identifier. Within each sub-directory, there are two components:

**Table 1:** Species collected for BiSID-5k^24^.

English name	Latin name	Geographical origin (state, country)	Year	No. of samples
Black grass	*Alopecurus myosuroides* L.	Hesse, Germany	2019	500
Common knotgrass	*Polygonum aviculare* L.	Baden-Wuerttemberg, Germany	2010	500
Common hemp-nettle	*Galeopsis tetrahit* L.	Rhineland-Palatinate, Germany	2021	500
Bistort	*Bistorta officinalis* L.	Baden-Wuerttemberg, Germany	2021	500
Red campion	*Silene dioica* L.	Bavaria, Germany	2021	500
Red clover	*Trifolium pratense* L.	Baden-Wuerttemberg, Germany	2021	500
Herb robert	*Geranium robertianum* L.	Bavaria, Germany	2021	500
Common chickweed	*Stellaria media* L.	Serbia	2021	500
Meadow foxtail	*Alopecurus pratensis* L.	Hesse, Germany	2021	500
Winter Oilseed Rape	*Brassica napus* L.	Occitanie, France	2022	500

The list includes number of samples, geographical origin and year of harvest for each species.

an HS_Raw.zip archive containing the unprocessed hyperspectral image (*HsScan.bil*) and its corresponding header file (*HsScan.bil.hdr*) and

an unprocessed RGB image (*RGB_Raw.jpg*) in JPEG format.

The header files contain metadata about acquisition conditions and wavelength allocation for the spectral bands. In addition to the bimodal pairs of seed images, we included 10 bimodal pairs of empty seed trays.

## Technical Validation

### Multimodal feature extraction

**Fig. 7:**
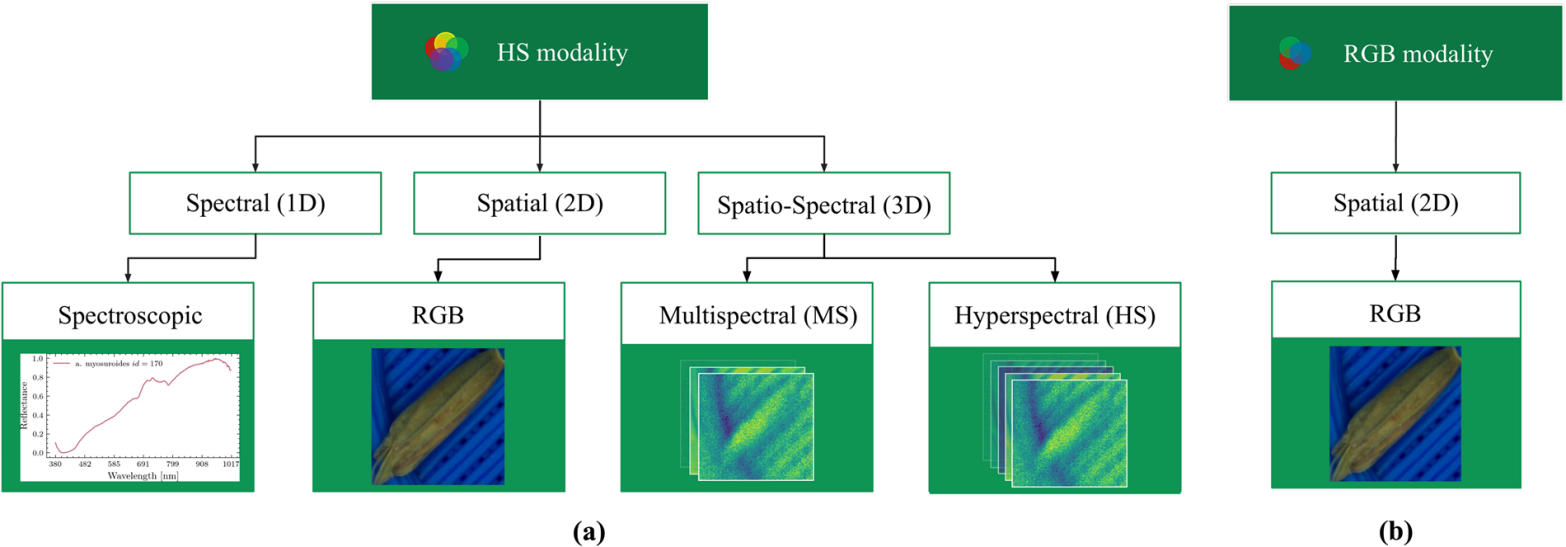
Overview of feature extraction methods for (**a**) HS and (**b**) RGB modalities.

There are multiple ways of how to extract and which features to extract from rgb and hyperspectral data (see Fig. 7). In our work we employ distinct feature extraction techniques for the RGB and hyperspectral modalities, tailored to the specific characteristics of each data type. For RGB imagery, feature extraction is inherently integrated into the classification ConvNet model. The model learns and extracts hierarchical feature representations in a data-driven manner during training, eliminating the need for a separate feature extraction step. As opposed to the RGB modality, we explored three different approaches for feature extraction from the hyperspectral modality and its utilisation:

**Fig. 8:**
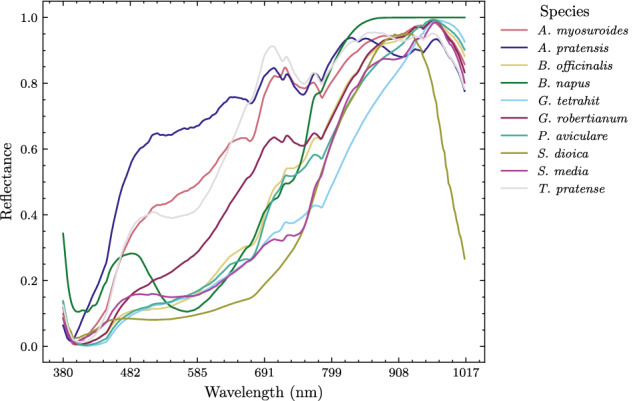
Average spectra for species in the dataset. The spectra were computed by extracting a central 5 × 5 region from each hyperspectral cube and averaging along the spatial dimensions, resulting in a 1 × 1 × 300 vector. This process was repeated for all 500 samples of each species and the results were averaged species-wise.

1. Firstly, we adopted a lightweight spectral approach commonly used in spectroscopy for feature extraction. From each hyperspectral cube (128 × 128 × 300), we extracted a central region of interest (ROI) of size 5 × 5 × 300. Subsequently, we applied mean pooling along the spectral dimension, resulting in feature vectors of size 1 × 300 (see Fig. 8). This approach preserves spectral information while sacrificing spatial details. We call the new modality *spectral*.
2. Secondly, we followed a computationally more complex spatio-spectral approach as well. Since most spectral bands are highly correlated, we opted to subsample every *n*-th spectral band from the hyperspectral cube. This reduces to some extent computational complexity while preserving large proportion of spectral and spatial features of the seeds. We call the new modality *multispectral (MS)*.
3. Lastly, we utilised the full hyperspectral cubes without any modification for Single-Label Image Classification^42^ or Whole-Image Classification^43^. This approach distinguishes our work from Multi-Label or Pixelwise Classification, which is commonly used in remote sensing^44–46^.

### Experimental setup and results

#### Baselines

To evaluate the performance of each feature extraction method described in the previous section, we established dedicated baselines tailored to each data modality.

##### RGB baselines

We utilised following variants of the ResNet^47^ for RGB images:

- 2D-ResNet-18 (2D-R18),
- 2D-ResNet-34 (2D-R34) and
- 2D-ResNet-50 (2D-R50).

##### Hyperspectral and multispectral baselines

Similarly to RGB baselines, we employed 3D versions of the ResNet for the both hyperspectral (HS) and newly introduced multispectral (MS) modalities:

3D-ResNet-18 (3D-R18),

3D-ResNet-34 (3D-R34) and

3D-ResNet-50 (3D-R50).

For HS data, we used the raw data with all 300 spectral bands, which fully utilise the spatio-spectral information from the hyperspectral cubes. Due to the continuous nature of hyperspectral spectra, adjacent wavebands exhibit high correlation, as they capture nearly identical information with only slight variations in spectral response. To simulate situations, where loading entire hyperspectral cubes into GPU memory is not feasible due to computational challenges and this redundancy in spectral information, we applied subsampling strategies to reduce data complexity. Specifically, we tested two interval-based wavebands subsampling approaches for MS modality:

*Step30*: Selecting every 30th spectral band, covering the visible and near-infrared (VNIR) range and

*Step60*: Selecting every 60th spectral band, also representing the VNIR range

Moreover, we varied spatial resolution of hyperspectral and multispectral cubes as well. In particular, we employed set of three different spatial sizes, namely by resizing original 128 × 128 data cubes to

32 × 32,

64 × 64 and

96 × 96.

##### Spectral baselines

Ultimately, we adopted supervised approaches for the spectral data, tailored to our experimental setup and inspired by the previous study^48^. The following models were used:

- Logistic Regression (LR),
- Decision Tree (DT),
- Random Forest (RF) and
- Multilayer Perceptron (MLP)

#### Training settings

Both RGB and MS baselines were trained for 50 epochs utilising AdamW^49^. For smaller 2D-ResNets learning rate of 1 × 10^−5^ was applied, whereas for larger 3D-ResNet we used higher learning rate of 1 × 10^−4^. The uniform weight decay of 4 × 10^−3^ was chosen for both types of models. The beta parameters (*β*_1_, *β*_2_) of optimiser were set to 0.9 and 0.999 respectively. During training, we used a distributed batch size of 64. For deep ResNets, we employ a train/validation/test split of the paired images, allocating 70% for training, 15% for validation and 15% for testing. For the shallow machine learning (ML) models, we used the default hyperparameter values provided by the scikit-learn package and applied a 70/30 train-test split. We assessed the performance of the models using standard metrics, including

Accuracy,

Precision,

Recall

and F1-Score.

(see Eqs. (1) – (4)). We averaged the results over three independent runs.

1 $$Accuracy=\frac{TP+TN}{TP+TN+FP+FN}$$

2 $$Precision=\frac{TP}{TP+FP}$$

3 $$Recall=\frac{TP}{TP+FN}$$

4 $$F1{\rm{ \mbox{-} }}Score=\frac{2TP}{2TP+FP+FN}$$

*where*:

- *TP* is True Positives,
- *TN* is True Negatives,
- *FP* is False Positive,
- *FN* is False Negatives;

All experiments were conducted on a workstation equipped with an AMD Ryzen Threadripper 3970X 32-Core Processor, 64 GB of RAM and 2× Nvidia RTX A6000, each with 48 GB of memory. Models for RGB, MS and HS modalities have been created using Keras 3^50^ with TensorFlow^51^ as backend, whereas spectral approaches used scikit-learn^52^.

#### Results

As shown in Table 2, ResNet models trained on the MS modality achieve the highest performance. The smallest model, 3D-R18, trained on 10 spectral bands (*Step30*), reaches the highest overall accuracy of 99.60%, slightly surpassing the larger 3D-R34 (99.55%) and 3D-R50 (99.20%). A similar trend is observed across all metrics: 3D-R18 achieves the highest Precision (99.62%), Recall (99.61%) and F1-score (99.60%), followed closely by 3D-R34 and 3D-R50.

**Table 2:** Results for the evaluated models, averaged over three independent runs.

Model	Modality	Sample shape	Learning rate	Batch size	Accuracy	Precision	Recall	F1-Score	Params (M)
LR	Spectral	1 × 300	—	—	90.67 ± 0.48	90.87 ± 0.42	90.67 ± 0.48	90.60 ± 0.48	—
DT	Spectral	1 × 300	—	—	89.13 ± 0.38	89.20 ± 0.44	89.13 ± 0.38	89.12 ± 0.38	—
RF	Spectral	1 × 300	—	—	94.62 ± 0.64	94.67 ± 0.60	94.62 ± 0.64	94.59 ± 0.63	—
MLP	Spectral	1 × 300	1 × 10^−3^	—	95.29 ± 0.55	95.42 ± 0.48	95.29 ± 0.55	95.30 ± 0.54	—
2D-R18	RGB	192 × 192 × 3	1 × 10^−5^	64	98.62 ± 0.22	98.60 ± 0.23	98.67 ± 0.21	98.62 ± 0.23	11.192
2D-R34	RGB	192 × 192 × 3	1 × 10^−5^	64	97.59 ± 0.10	97.59 ± 0.11	97.68 ± 0.11	97.59 ± 0.11	21.307
2D-R50	RGB	192 × 192 × 3	1 × 10^−5^	64	96.22 ± 0.49	96.2 ± 0.50	96.28 ± 0.51	96.19 ± 0.50	38.505
3D-R18	MS	96 × 96 × 10 × 1	1 × 10^−4^	64	**99.60 ± 0.11**	**99.62 ± 0.11**	**99.61 ± 0.10**	**99.60 ± 0.11**	33.175
3D-R34	MS	96 × 96 × 10 × 1	1 × 10^−4^	64	99.55 ± 0.17	99.58 ± 0.16	99.57 ± 0.15	99.57 ± 0.16	63.492
3D-R50	MS	96 × 96 × 10 × 1	1 × 10^−4^	64	99.20 ± 0.11	99.23 ± 0.12	99.19 ± 0.11	99.21 ± 0.11	115.047
3D-R18	HS	96 × 96 × 300 × 1	1 × 10^−4^	64	98.89 ± 0.28	98.94 ± 0.27	98.93 ± 0.27	98.92 ± 0.28	33.175
3D-R34	HS	96 × 96 × 300 × 1	1 × 10^−4^	64	98.76 ± 0.28	98.81 ± 0.26	98.79 ± 0.25	98.78 ± 0.27	63.492
3D-R50	HS	96 × 96 × 300 × 1	1 × 10^−4^	64	98.53 ± 0.29	98.59 ± 0.30	98.57 ± 0.29	98.57 ± 0.29	115.047

ResNets trained on the HS modality perform  ~ 1% worse across all metrics compared to those trained on MS data. This indicates that increasing the number of spectral bands does not necessarily improve performance. Nevertheless, ResNets trained on HS cubes still outperform models trained on the RGB images by an average margin of 0.3–2.3%, depending on model size.

A consistent pattern emerges across all ResNets: smaller architectures tend to achieve better performance. This could be attributed to the relatively small dataset size or suboptimal hyperparameter values (e.g., number of training epochs, learning rate, or weight decay). Larger models generally require higher computational budgets or higher learning rate schedules to reach optimal results. Notably, the impact of model size is particularly pronounced for the RGB modality, where the performance gap between the best-performing 2D-R18 (98.62%) and the worst-performing 2D-R50 (96.22%) is the most significant.

In contrast, traditional ML approaches trained on spectral data perform substantially worse. Among them, MLP achieves the highest accuracy (95.29%), while DT exhibits the weakest performance, reaching only  ~ 89% across all metrics. LR slightly outperforms DT with an accuracy of 90.67%.

##### Ablation study: investigating the impact of spectral subsampling and spatial resolution

The Table 3 presents scenarios in which the spatial and spectral dimensions of the data cube — either independently or in combination—are manipulated to assess their impact on model performance. The results indicate that training on entire hyperspectral cubes with 300 spectral bands yields the best performance only when the spatial resolution is relatively low. In such cases, ResNets trained on the MS modality cannot fully exploit spatial features, which makes spectral resolution the dominant factor. For example, ResNets trained on HS data with a spatial resolution of 32 × 32 outperform those trained on MS cubes by approximately 0.7–1.0% across all metrics. A similar trend is observed for multispectral ResNets: models trained on 10 spectral bands outperform those trained on only 5 bands. However, as the spatial resolution increases, ResNets trained on MS cubes start to surpass those trained on HS data, indicating a shift in the balance between spectral and spatial information.

**Table 3:** Results of the 3D-ResNet models across different spatial and spectral resolutions for multispectral (MS) and hyperspectral (HS) modalities.

Model	Modality	Spatial resolution	Spectral subsampling	Batch size	Accuracy	Precision	Recall	F1-Score
3D-ResNet-18	MS	32 × 32	Step60	64	97.07 ± 0.29	97.16 ± 0.28	97.12 ± 0.30	97.13 ± 0.29
	MS	32 × 32	Step30	64	97.56 ± 0.12	97.63 ± 0.14	97.57 ± 0.13	97.59 ± 0.14
	HS	32 × 32	—	64	98.27 ± 0.22	98.34 ± 0.23	98.27 ± 0.22	98.29 ± 0.23
	MS	64 × 64	Step60	64	98.22 ± 0.07	98.29 ± 0.08	98.23 ± 0.06	98.24 ± 0.07
	MS	64 × 64	Step30	64	99.02 ± 0.27	99.08 ± 0.27	99.02 ± 0.28	99.04 ± 0.27
	HS	64 × 64	—	64	97.60 ± 0.11	97.69 ± 0.11	97.61 ± 0.12	97.63 ± 0.11
	MS	96 × 96	Step60	64	99.11 ± 0.13	99.17 ± 0.13	99.09 ± 0.12	99.12 ± 0.12
	MS	96 × 96	Step30	64	**99.60** ± **0.11**	**99.62** ± **0.11**	**99.61** ± **0.10**	**99.60** ± **0.11**
	HS	96 × 96	—	64	98.89 ± 0.28	98.94 ± 0.27	98.93 ± 0.27	98.92 ± 0.28
3D-ResNet-34	MS	32 × 32	Step60	64	97.20 ± 0.65	97.27 ± 0.65	97.23 ± 0.64	97.24 ± 0.65
	MS	32 × 32	Step30	64	97.91 ± 0.45	98.02 ± 0.44	97.92 ± 0.43	97.94 ± 0.45
	HS	32 × 32	—	64	98.04 ± 0.33	98.10 ± 0.33	98.05 ± 0.33	98.06 ± 0.33
	MS	64 × 64	Step60	64	98.36 ± 0.28	98.45 ± 0.27	98.36 ± 0.25	98.39 ± 0.26
	MS	64 × 64	Step30	64	98.53 ± 0.29	98.60 ± 0.29	98.55 ± 0.29	98.56 ± 0.29
	HS	64 × 64	—	64	98.31 ± 0.50	98.40 ± 0.49	98.33 ± 0.53	98.34 ± 0.50
	MS	96 × 96	Step60	64	98.76 ± 0.38	98.81 ± 0.36	98.73 ± 0.39	98.77 ± 0.38
	MS	96 × 96	Step30	64	99.55 ± 0.17	99.58 ± 0.16	99.57 ± 0.15	99.57 ± 0.16
	HS	96 × 96	—	64	98.76 ± 0.28	98.81 ± 0.26	98.79 ± 0.25	98.78 ± 0.27
3D-ResNet-50	MS	32 × 32	Step60	64	96.84 ± 0.35	96.95 ± 0.37	96.88 ± 0.31	96.89 ± 0.34
	MS	32 × 32	Step30	64	97.73 ± 0.11	97.81 ± 0.07	97.73 ± 0.13	97.76 ± 0.10
	HS	32 × 32	—	64	97.78 ± 0.45	97.86 ± 0.45	97.78 ± 0.46	97.80 ± 0.46
	MS	64 × 64	Step60	64	98.44 ± 0.12	98.50 ± 0.10	98.46 ± 0.15	98.47 ± 0.12
	MS	64 × 64	Step30	64	98.71 ± 0.44	98.75 ± 0.46	98.72 ± 0.42	98.72 ± 0.44
	HS	64 × 64	—	64	98.00 ± 0.29	98.06 ± 0.28	98.01 ± 0.29	98.02 ± 0.28
	MS	96 × 96	Step60	64	99.20 ± 0.11	99.23 ± 0.12	99.19 ± 0.11	99.21 ± 0.11
	MS	96 × 96	Step30	64	99.56 ± 0.23	99.57 ± 0.22	99.56 ± 0.22	99.56 ± 0.22
	HS	96 × 96	—	64	98.53 ± 0.29	98.59 ± 0.30	98.57 ± 0.29	98.57 ± 0.29

For MS, *Step60* refers to selecting every 60th spectral band (5 spectral bands from the 300-band hyperspectral cube), while *Step30* refers to selecting every 30th spectral band (10 spectral bands). Note that for the HS modality, no spectral subsampling was applied and all 300 spectral bands were used. The results for the highest scores across all metrics are shown in **bold**. All results were averaged over three independent runs.

Conversely, using too few spectral bands also leads to a performance decline, suggesting the existence of an optimal trade-off—a “sweet spot”—between spectral and spatial resolution, where the model achieves peak performance. Our experiments confirm that this pattern holds independently of model size. Specifically, ResNets trained using the *Step30* subsampling strategy with a spatial resolution of either 64 × 64 or 96 × 96 achieve the best results, yielding:

0.2–1.4% improvement over models trained on entire hyperspectral cubes and

0.2–0.8% improvement compared to those trained with the *Step60* subsampling strategy.

However, it remains unclear whether *Step30* represents the absolute optimal subsampling strategy or if increasing the number of spectral bands (>10) could further enhance performance.

In contrast to spectral subsampling, the effect of spatial resolution is more straightforward. Regardless of the subsampling strategy, increasing the spatial resolution of multispectral or hyperspectral images consistently improves the classification performance of the ResNet models.

## Usage Notes

The BiSID-5k dataset^24^ represents the largest open-access whole-image bimodal dataset for seed classification. Its scale and diversity make it a valuable resource for the AI community to develop advanced classification systems (i.a. for seed production or for meeting certification requirements set by regulatory agencies). Except the data itself, we benchmark the dataset against the state-of-the-art (SOTA) model under different scenarios, demonstrating the flexibility and versatility of the data use. We are confident that datasets like BiSID-5k^24^ will advance computer vision methodologies for hyperspectral imaging, facilitating the development of novel architectures and advancing the field. The dataset will be valuable for researchers studying spectral and morphological properties of seeds, as well as regulatory authorities seeking to improve quality assessment procedures.

### Future multimodal applications

The dataset includes four types of modalities—hyperspectral, multispectral, spectroscopic and RGB, which are either directly present or easily derivable. The integration of these modalities in multimodal scenarios has gathered increasing interest in recent years. While this work does not compare existing multimodal baselines, it provides a comprehensive review of the relevant literature. For instance, it could be feasible to apply similar approach^53^, where the authors extracted morphological, textural and spectral features from hyperspectral cubes and fuse these features together for classification of rice varieties. The more recent study^42^ investigated the performance of a pretrained self-supervised bimodal masked autoencoder (BiMAE) for seed classification. The study involved pretraining BiMAE on RGB and hyperspectral data on 19 different seed species, followed by finetuning on multispectral and RGB data, which showcases its adaptability across multiple modalities.

## Data Availability

The dataset is publicly available at 10.25532/OPARA-810 under the Creative Commons Attribution (CC BY) license.

## Code availability

We provide all the necessary scripts for the entire pipeline, from preprocessing to model training and evaluation. This includes a script for segmenting individual seeds from the seed tray using SAM and a script for converting the bimodal dataset into the TFRecord format (see https://www.tensorflow.org/tutorials/load_data/tfrecord), which is particularly beneficial for large datasets like ours. This format improves I/O throughput during training and integrates seamlessly with TensorFlow pipelines, enabling faster and more efficient data loading and augmentation. Additionally, we share scripts for training and evaluating all evaluated models. The code is developed using Python 3.10 and is accessible at https://github.com/max-kuk/bisid-5k-tools. The repository includes a requirements.txt file specifying all necessary packages along with their versions. Additional information about the dataset is available at https://max-kuk.github.io/bisid-5k.
