# Correction:A bimodal image dataset for seed classification from the visible and near-infrared spectrum

**DOI:** 10.1038/s41597-026-06771-w

**Published:** 2026-02-06

**Authors:** Maksim Kukushkin, Martin Bogdan, Simon Goertz, Jan-Ole Callsen, Eric Oldenburg, Matthias Enders, Thomas Schmid

**Affiliations:** 1 Martin Luther University Halle-Wittenberg, Halle(Saale), 06108 Germany; 2 Leipzig University, Leipzig, 04109 Germany; 3 NPZ Innovation GmbH, Holtsee, 24363 Germany; 4 Norddeutsche Pflanzenzucht Hans-Georg Lembke KG, Hohenlieth-Hof 1, 24363 Holtsee, Germany; 5 Lancaster University in Leipzig, Leipzig, 04109 Germany

Correction to: *Scientific Data* 10.1038/s41597-025-05979-6, published online 8 October 2025.

In the version of this article initially published, in six instances, inline list items (e.g., (i)–(iii)) were formatted incorrectly and are now amended in the HTML and PDF versions of the article.

